# Structural Aspects of the Formation of Multilayer Composites from Dissimilar Materials upon High-Pressure Torsion

**DOI:** 10.3390/ma16103849

**Published:** 2023-05-19

**Authors:** Roman Sundeev, Anna Shalimova, Stanislav Rogachev, Olga Chernogorova, Alexander Glezer, Alexey Ovcharov, Igor Karateev, Natalia Tabachkova

**Affiliations:** 1 Department of Nanoelectronics, MIREA—Russian Technological University, Vernadskogo Prospect, 78, 119454 Moscow, Russia; 2 I.P. Bardin Science Institute for Ferrous Metallurgy, Radio Street, 22/9, 105005 Moscow, Russia; shalimanna@yandex.ru (A.S.); a.glezer@mail.ru (A.G.); 3 Department of Physical Metallurgy and Physics of Strength, National University of Science and Technology “MISiS”, Leninski Prospect, 4, 119049 Moscow, Russia; csaap@mail.ru (S.R.); ntabachkova@gmail.com (N.T.); 4 Baikov Institute of Metallurgy and Materials Science RAS, Leninski Prospect, 49, 119334 Moscow, Russia; olga100748@mail.ru; 5 National Research Centre “Kurchatov Institute”, Akademika Kurchatova Square, 1, 123182 Moscow, Russia; ovcharov.91@gmail.com (A.O.); iakarateev@gmail.com (I.K.)

**Keywords:** composite, phase transformation, high-pressure torsion, severe plastic deformation, layered structures

## Abstract

A multi-metal composite was consolidated from the Ti_50_Ni_25_Cu_25_ and Fe_50_Ni_33_B_17_ alloys by room-temperature high-pressure torsion (HPT). The structural research methods used in this study were X-ray diffractometry, high-resolution transmission electron microscopy, scanning electron microscopy with an electron microprobe analyzer in the mode of backscattered electrons, and the measurement of indentation hardness and modulus of the composite constituents. The structural aspects of the bonding process have been examined. The method of joining materials using their coupled severe plastic deformation has been established to play a leading role in the consolidation of the dissimilar layers upon HPT.

## 1. Introduction

Layered cermet and multilayer multimetallic composites (MMC) are the most important class of functional materials that represent a wide range and unique combination of valuable properties such as high strength, corrosion resistance, electrical and thermal conductivity, heat resistance, and wear resistance. In particular, MMC composed of crystalline metals and alloys is characterized by high magnetic, electromagnetic, and mechanical properties that surpass those of the original precursors. Methods for obtaining such composites and their service properties have been extensively studied [1,2,3,4,5,6].

Naturally, it is more efficient to use layered materials for the study of the structure of interfaces between precursors and the phase transitions upon the preparation of composites and their further operation. In such materials, the lengths of the precursor laminates and boundary regions (at least in the initial material) can be significant. Based on this assumption, the transformation, structure, and properties of a naturally layered amorphous–crystalline Ti_2_NiCu composite upon high-pressure torsion (HPT) were earlier studied in detail [7]. The method of severe plastic deformation by HPT is characterized by critical loads, which make it possible to reach the limit of grain structure refinement in the sample. It is an effective method to attain grain sizes of 100 nm or less [8,9,10,11], even in hard-to-deform metals and intermetallic compounds [12,13]. The grain refinement, in the first approximation, introduces new obstacles to dislocation motion into the structure (grain boundaries of different natures, triple junctions) and, thus, leads to the strengthening of the material. It is also well known that such deformation initiates phase transformations [14,15,16,17]. In this regard, HPT now is used to prepare nanocomposites and hybrid materials [18,19,20]. Naturally, upon HPT, the state of the heterogeneous precursor changes, and a hybrid structure is formed in the composites prepared by such methods. Modern hybrid materials with unique properties are typically synthesized by HPT from nanocrystalline materials with different parent structures [21,22,23,24]. An extensive review devoted to the study of the relationship between the preparation conditions, microstructure, and mechanical properties of modern hybrid materials formed by the HPT method from crystalline dissimilar materials is presented in [25]. It is shown that, in this case, the initial layers upon HPT are fragmented. As usual, the final structure is a mixture of nanoscale fragments of the initial structural constituents. Various structural defects, such as interphase interfaces, grain boundaries, dislocation arrangements, and discontinuities of various types in such composites, lead to an enhancement of their mechanical properties. However, there are virtually no published papers on the preparation of composites from layers of rapidly quenched metallic amorphous and amorphous/crystalline materials, in which, as is proven by the experimental data, the consolidation of amorphous layers is associated with interfacial interaction in thin boundary regions [26,27]. In this case, the layers differing not only in chemical composition but also in topology should be consolidated. Studies show that an important role is played by the structure of parent layers [28] and by the structure of the transition regions between such layers [29]. Usually, the melt-quenched and nanocrystalline layers chosen for the composite preparation during HPT exhibit various transformations, and the unknown structure states formed at transition regions can affect the processes of composite formation.

The aim of this paper is to study the structural aspects of MMC formation from topologically dissimilar metal layers of the Ti_50_Ni_25_Cu_25_ and Fe_50_Ni_33_B_17_ alloys in the mode of increasing the degree of deformation upon HPT. It should be noted that the individual precursors behave differently under the same HPT conditions. The Ti_50_Ni_25_Cu_25_ alloy undergoes a phase transformation from a nanocrystalline to an amorphous state, whereas the other precursor alloy, Fe_50_Ni_33_B_17_, on the contrary, passes from an amorphous to a crystalline state. This study includes not only an analysis of the structural evolution in the deformed precursor layers but also a clarification of the effect of transition zones between the layers on the degree of composite consolidation. It is also proposed to estimate the degree of cooperative effect of structural changes in different layers on the degree of consolidation upon joint deformation. Such a systematic structural study has been carried out for the first time.

## 2. Materials and Methods

### 2.1. Materials

The constituent layers for the future MMC were prepared by melt quenching. The Fe_50_Ni_33_B_17_ alloy was melted in a vacuum induction furnace (Balzers Inc., MI, USA). The amorphous Fe_50_Ni_33_B_17_ ribbons were melt spun from round rods 6–8 mm in diameter and 300–400 mm in length. The rods were prepared by drawing the melt into quartz tubes; the melt temperature was not substantially higher than the solidification temperature of this alloy. Such requirements are caused by the intense development of porosity and oxidation upon melt overheating. Then, the rods were cut into pieces of about 50 g in weight. Stainless steel and copper with nickel and chromium coatings were used as materials for the quenching disk upon spinning. The nozzle slot width was 1.2 mm, the rotation speed of the quenching disk was 2200 rpm, and the cooling rate was ≈10^6^ K/s. The finished Fe_50_Ni_33_B_17_ alloy ribbon was 10 mm wide and 20 µm thick.

The Ti_50_Ni_25_Cu_25_ ribbons were prepared in an amorphous state using the single-roll melt quenching (MQ) method. Alloy ingots were initially prepared from high-purity nickel, titanium, and copper with six remeltings in an arc furnace in an argon atmosphere. The preforms obtained were melted in a quartz crucible in a helium atmosphere and extruded through a narrow nozzle in the crucible onto the surface of a rotating copper disk. The cooling rate was 10^6^ K/s. The initial ribbon was on average 46 ± 1 µm thick. Then, the amorphous ribbon was annealed at 500 °C for 30 min in the air to achieve a crystalline state.

### 2.2. Material Preparation

A sandwich formed from three ribbons, such as one amorphous Fe_50_Ni_33_B_17_ alloy ribbon between two crystalline Ti_50_Ni_25_Cu_25_ alloy ribbons, was treated by HPT at a pressure of 6 GPa in flat anvils. Such three-layer samples allowed us to study the structural evolution of the precursors during the consolidation of MMC in the mode of increasing the degree of mutual deformation by HPT. The sample examination was performed directly in all the layers and in the transition regions. Earlier, it was demonstrated that one of the precursors (Ti_50_Ni_25_Cu_25_) upon HPT undergoes a phase transformation from a crystalline to an amorphous state [30]. The other precursor underwent a phase transformation from an amorphous to a crystalline state under the same HPT conditions [31].

### 2.3. Experimental Methods

The first series of blanks were subjected to compressive deformation in flat anvils without shear. The exposure times under pressure were 1, 2, 5, 8, 16, and 25 min, which corresponded to the times of the HPT tests of the samples. The second series of samples was subjected to HPT to 1, 2, 5, 8, 16, and 25 revolutions (*n*) of the movable anvil at a rotation speed of 1 rpm.

The structural phase transformations and mechanical properties were traced on the samples deformed by HPT to all degrees of deformation (*n* = 2, 5, 8, 16, and 25).

All structural studies, except for the examination of phase transformations on the outer MMC surfaces, were carried out on cross-sections, which were prepared according to the procedure described in [32].

Both MMC sample surfaces were studied by X-ray diffraction (XRD) analysis with a DRON-3M (Bourevestnik JSC, St. Petersburg, Russia) diffractometer according to the Bragg–Brentano method in a stepwise mode with CoKα radiation using a graphite monochromator on a diffracted beam.

A JSM-IT500 (JEOL Ltd., Tokyo, Japan) scanning electron microscope (SEM) with an electron microprobe analyzer in backscattered electron mode at magnifications of 300 and 800 was used to examine the cross-sections of the MMC samples.

A SHIMADZU DUH-211/DUH-211S (Shimadzu Corporation, Kyoto, Japan) ultra-micro hardness tester was used to measure the distribution of indentation hardness (*H*_IT_) and indentation modulus (*E*_IT_) [33] over MMC samples.

A Titan 80–300 (Thermo Fisher Scientific, Waltham, MA, USA) (scanning) high-resolution transmission electron microscope ((S)TEM) equipped with a spherical aberration corrector (Cs-corrector), a high-angle annular dark-field detector (HAADF), and JEM-2100 (JEOL Ltd., Tokyo, Japan) with an X-ray microanalyzer was used at an accelerating voltage of 300 kV to study the samples using transmission electron microscopy (HRTEM). The samples for the HRTEM examination were prepared from selected positions (usually in the middle of the sample radius) of the cross-sections of the tested sample using the focused ion beam technique. 

## 3. Results

### 3.1. X-ray Diffraction

**Figure 1:**
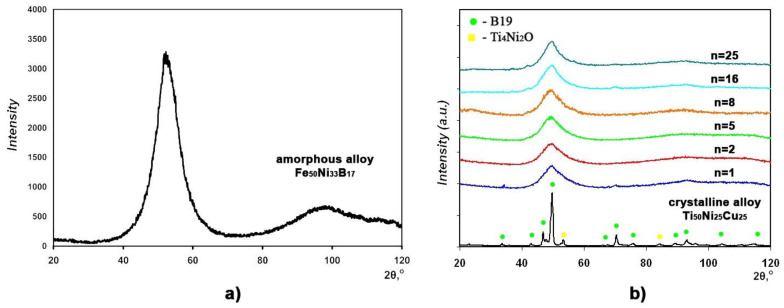
XRD patterns of (**a**) Fe_50_Ni_33_B_17_ amorphous alloy and (**b**) initial Ti_50_Ni_25_Cu_25_ crystalline alloy and the outer MMC layers (Ti_50_Ni_25_Cu_25_) after HPT.

The XRD spectra of the Fe_50_Ni_33_B_17_ amorphous alloy and the crystallized Ti_50_Ni_25_Cu_25_ alloy in the initial states are shown in Figure 1. The XRD pattern of the Fe_50_Ni_33_B_17_ alloy exhibits only two halos (Figure 1a), which are typical of an amorphous state, whereas the initial crystalline state of the Fe_50_Ni_33_B_17_ alloy (Figure 1b) is represented by mainly the B19 phase and a small quantity of the Ti_4_Ni_2_O phase. The HPT behavior of the initial precursors used for consolidating the MMC sample was studied in [30,31]. No consolidation of the parent ribbons was found in the MMC samples subjected only to compression without shear. A similar result was observed for samples after HPT to *n* = 1. The XRD patterns of the outer layers allowed us to conclude that amorphization of the outer, initially crystalline, Ti_50_Ni_25_Cu_25_ layers of the MMC alloy began to develop upon HPT to *n* > 1 (Figure 1b).

### 3.2. Scanning Electron Microscopy 

**Figure 2:**
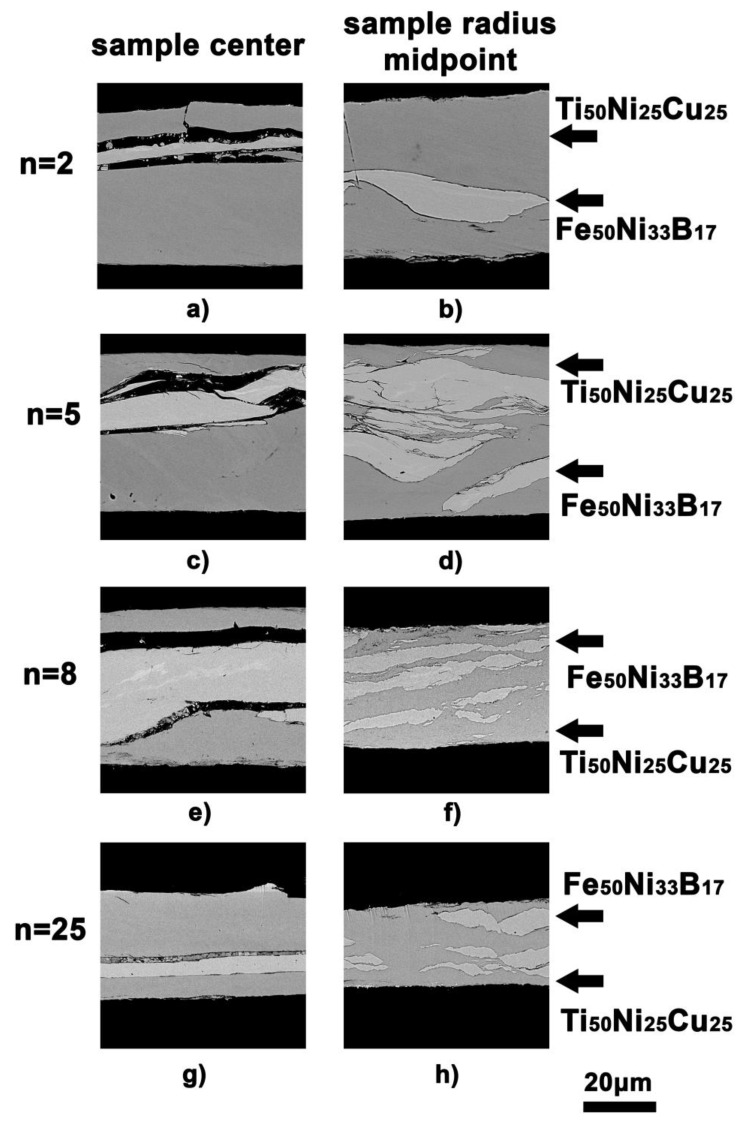
SEM images of the MMC cross-section structure after HPT: sample centre (**a**) *n* = 2; (**c**) *n* = 5; (**e**) *n* = 8; (**g**) *n* = 25 and sample radius midpoint (**b**) *n* = 2; (**d**) *n* = 5; (**f**) *n* = 8; (**h**) *n* = 25.

The structural changes in the consolidated MMC were examined using SEM and TEM. Figure 2 shows the SEM images of the MMC structure in the center of the sample and in the middle of the sample radius as a function of the degree of deformation by HPT.

The evolution of the mutual arrangement of the precursor layers upon deformation at *n* >2 is clearly seen in Figure 2a,b. Changes in the position and thickness of the layers can be caused by the deformation gradient along the sample radius. Such a gradient is characteristic of HPT. There was a noticeable difference in the mutual arrangement of the layers in the center of the sample and at the half-radius position (Figure 2a). The presence of cavities between the layers and cracks in the Ti_50_Ni_25_Cu_25_ layer shows a lack of consolidation in the central zone, whereas at the half-radius position of the same sample, the layers converge, and no cavities are observed. The Fe_50_Ni_33_B_17_ layer was bent, unevenly thinned, and refined (Figure 2b). Deformation to *n* = 5 and above also causes the mixing of the layers and the formation of multilayer structures at the edges of the disk sample (Figure 2c–h). After HPT to *n* = 8, the specific features of the structure were as follows: the consolidation in the center of the sample was poor, whereas, at the middle of the sample radius, the refinement of the Fe_50_Ni_33_B_17_ layer and mixing of small fragments of the Fe_50_Ni_33_B_17_ alloy with the Ti_50_Ni_25_Cu_25_ alloy were even more distinct (Figure 2f). After HPT to *n* = 25, there was a spacing between the layers in the sample center, which was filled with the fragments of the Fe_50_Ni_33_B_17_ and Ti_50_Ni_25_Cu_25_ alloys (Figure 2g). In the middle of the radius (Figure 2h), the multilayer configurations of the Fe_50_Ni_33_B_17_ alloy fragments were formed against the Ti_50_Ni_25_Cu_25_ background, and complete consolidation and mixing of the layers were observed.

### 3.3. High-Resolution Transmission Electron Microscopy

**Figure 3:**
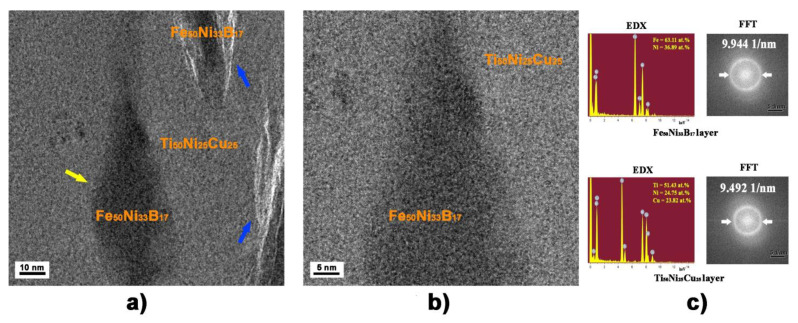
HRTEM image of MMC structure after HPT to *n* = 5: (**a**) two types of boundaries (transition regions) between heterogeneous MMC layers, (**b**) visually continuous transition regions between heterogeneous layers, and (**c**) FFT and EDX images of dissimilar MMC layers.

The TEM examination of the MMC structure showed various types of consolidated transition regions between the dissimilar layers subjected to HPT to *n* = 5 (Figure 3).

In some regions, loose transition zones 1–10 nm wide were formed between the layers (blue arrows in Figure 3a). In other regions (yellow arrow in Figure 3a) of the sample, the positions of consolidation can be found only by contrast in the TEM images because of the different scattering power of atoms in the layers that make up the MMC and by different halo widths in the Fourier transform images (Figure 3c). It is evident that the first diffuse haloes of the Fast Fourier transform (FFT) layers substantially differ in size. These correspond to the angular position of the first diffuse halo in the XRD pattern of the amorphous alloys used. The Energy dispersive X-ray (EDX) data shown in Figure 3c correctly confirm the local chemical composition on both sides of the boundary between the layers. As shown in Figure 3b, the amorphous phase (Fe_50_Ni_33_B_17_) continuously transits into the other amorphous phase (Ti_50_Ni_25_Cu_25_) without any pronounced transition zone. Analogous results were also observed for the samples subjected to HPT to *n* = 25.

### 3.4. Chemical Composition of MMC after HPT

The following questions arise: (1) Does the chemical composition of the initial alloys change during the mixing of the layers upon HPT, and (2) does the diffusion of atoms occur through the interface between the Fe_50_Ni_33_B_17_ fragments and the Ti_50_Ni_25_Cu_25_ matrix in the regions where the layers of different alloys are consolidated by HPT?

To clarify these issues, we studied both the qualitative and quantitative chemical compositions of the MMC layers using SEM examination with an X-ray electron microprobe analyzer in the backscattered electron mode.

**Figure 4:**
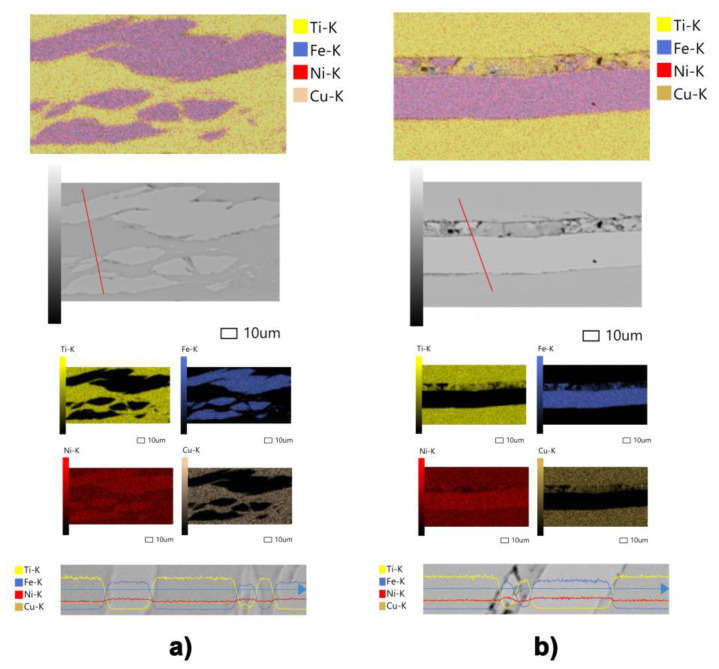
Distribution of chemical elements and microstructure of MMC after HPT to *n* = 25 over the cross-section of the sample with different types of interfaces: (**a**) complete consolidation of layers and (**b**) boundary between layers consisting of a mixture of fragments.

Qualitative and quantitative chemical analyses were carried out for all samples without exception. The results of all measurements were similar; therefore, the data were provided only for HPT to *n* = 25. As shown in Figure 4, the precursor materials after deformation virtually retained their initial average chemical composition. Quantitative measurements of the chemical composition in the layers of different alloys indicated, on average, constant ratios of elements in the precursor bands after deformation. Hence, it follows that the intermediate zones between the MMC layers prevent the diffusion of atoms from one layer of the composite to another.

### 3.5. Mechanical Properties

**Figure 5:**
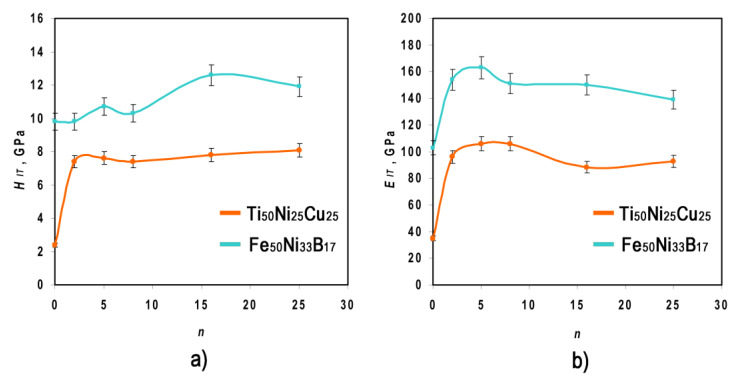
*H_IT_* (**a**) and *E_IT_* (**b**) as a function of *n* for the Ti_50_Ni_25_Cu_25_ and Fe_50_Ni_33_B_17_ layers.

The MMC structural states were also traced along the cross-section by analyzing the “load-unload” diagrams upon indentation. The indentation hardness (*H*_IT_) and indentation modulus (*E*_IT_) of the MMC were measured both in the initial state and after HPT (Figure 5).

It is seen that the *H*_IT_(*n*) dependences for the Ti_50_Ni_25_Cu_25_ and Fe_50_Ni_33_B_17_ layers were different. The *H*_IT_(*n*) curve for the Ti_50_Ni_25_Cu_25_ layer exhibited a kink at HPT to *n* = 2. An increase in the *H*_IT_ of the Ti_50_Ni_25_Cu_25_ layer was associated with the occurrence of the structural-phase “crystalline–amorphous state” transition in the layer upon HPT deformation corresponding to *n* = 2. On the contrary, *H*_IT_(*n*) for the Fe_50_Ni_33_B_17_ layer remained virtually unchanged with an increasing degree of deformation. The dependence of *E*_IT_ on the degree of deformation for both layers was similar.

The hardness-to-modulus ratio λ = *H*_IT_/*E*_IT_ [33] upon HPT varied between 0.07 and 0.09 for the Ti_50_Ni_25_Cu_25_ layer and between 0.09 and 0.10 for the Fe_50_Ni_33_B_17_ layer. The λ ratio serves as a qualitative comparative characteristic of the resistance of materials to deformation under mechanical loading and, therefore, reflects their structural state. According to the concepts reported in the literature [34,35], λ ≈ 0.05–0.09 corresponds to the amorphous-nanocrystalline state.

## 4. Discussion of Results

**Figure 6:**
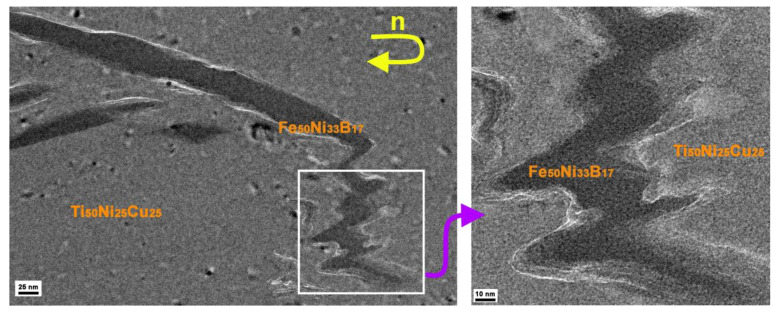
Changes in the shape of the initially rectangular profile of the Fe_50_Ni_33_B_17_ layer after HPT to *n* = 5.

The subject of the study in this paper was the three-layered MMC. The outer layers were from the crystallized Ti_50_Ni_25_Cu_25_ alloy with an initial hardness of 2.3 GPa, and the inner layer was from the Fe_50_Ni_33_B_17_ amorphous alloy with an initial hardness of 9.3 GPa. The individual precursors that make up the MMC under study differ in behavior upon HPT under similar conditions. For example, an individual Ti_50_Ni_25_Cu_25_ alloy upon HPT to *n* = 2–4 underwent a structural phase transformation from the crystalline to the amorphous state [30]. As shown in Figure 1b, the Ti_50_Ni_25_Cu_25_ alloy exhibits a similar behavior upon HPT of the MMC: at *n* = 2–4, it undergoes phase transformation into an amorphous state and is then deformed as an amorphous material. The Fe_50_Ni_33_B_17_ alloy underwent crystallization upon HPT already at *n* = 1 [31] and failed at a slight increase in deformation. Unlike the Ti_50_Ni_25_Cu_25_ alloy, the Fe_50_Ni_33_B_17_ alloy as a part of the MMC did not undergo any phase transformations upon HPT and remained amorphous up to a degree of deformation of *n* = 25. It is obvious that, starting from deformation to *n* ≥ 2 and up to *n* = 25, the Ti_50_Ni_25_Cu_25_ and Fe_50_Ni_33_B_17_ amorphous alloys were jointly deformed in MMC, and this is confirmed by the above hardness-to-modulus ratio λ. At the same time, the experimentally determined indentation moduli of the alloys differ by a factor of about 1.5. According to the high-resolution TEM data, HPT caused an uneven thinning of the harder amorphous Fe_50_Ni_33_B_17_ alloy layer, and this led to the formation of serrated boundary configurations (Figure 6).

With further increase in the degree of deformation, the serrated boundaries of the Fe_50_Ni_33_B_17_ alloy fragments were smoothed out, which was promoted by the shear component of the HPT. Fragments of the Fe_50_Ni_33_B_17_ alloy were refined, turned, and mixed with the Ti_50_Ni_25_Cu_25_ alloy, forming multilayer configurations (Figure 2b,d). No precipitation of any crystalline phases was observed in this alloy upon HPT to even more severe deformation (*e* = 9.2) (Figure 3). The more severe the deformation and the longer the distance from the sample center, the more intense the mixing of layer fragments. Our results of the *E*_IT_ measurements show that the layers substantially differed in plasticity, and therefore, the more ductile Ti_50_Ni_25_Cu_25_ amorphous alloy consumed most of the deformation. The Ti_50_Ni_25_Cu_25_ amorphous layers enveloped the Fe_50_Ni_33_B_17_ amorphous alloy fragments. Crystallization processes in such fragments were suppressed, and the Fe_50_Ni_33_B_17_ amorphous phase underwent only densification. This is indicated by the behavior of its indentation modulus *E_IT_*, which increased at the early stages of deformation, when the layers were not yet fully consolidated, and subsequently remained (within the error) virtually unchanged (Figure 5).

The shear stress upon HPT of the amorphous Ti_50_Ni_25_Cu_25_ alloy was previously determined experimentally [30] to be 570–580 MPa. For the Fe_50_Ni_33_B_17_ amorphous alloy, tensile strength was estimated to be 860–870 MPa with an allowance for the relationship between the indentation moduli *E*_IT_ of the Ti_50_Ni_25_Cu_25_ and Fe_50_Ni_33_B_17_ alloys.

A comparison of the shear stresses suggests that the consolidated layers differ in the deformation rate. The harder and stronger Fe_50_Ni_33_B_17_ layer delaminates, bends, and hinders the development of plastic deformation in the Ti_50_Ni_25_Cu_25_ layer [29]. The deformation turbulence of the composite generates stresses on the irregularities of the hard phase layer. The chains of the hard phase are destroyed, and the serrated boundaries of the Fe_50_Ni_33_B_17_ fragments are smoothed out. Fine fragments of the hard phase are redistributed and incorporated into the softer phase, forming multilayer configurations with continuous boundaries.

The consolidation of the two dissimilar amorphous alloys was recorded upon HPT. At least two types of transition regions between heterogeneous amorphous layers were observed at the sites of consolidation (Figure 3a): (1) a loose boundary zone of 1–10 nm in size and (2) a very narrow almost invisible transition region. On the basis of the polycluster model [36] of an amorphous state, which is characterized by a set of clusters composed of atoms corresponding to a chosen chemical composition, it can be assumed that the continuous structure of the transition zone between heterogeneous amorphous phases should contain clusters with variable compositions of atoms entering both phases. According to the Landau-Lifshitz theory of phase transitions [37], the chemical order parameter changes in the transition region between the layers. However, no changes in the chemical compositions of the Ti_50_Ni_25_Cu_25_ and Fe_50_Ni_33_B_17_ layers in the MMC were recorded experimentally (within the resolution of the method) after deformation. We also failed to record changes in the chemical compositions at the boundary itself, but this could be caused by the small thickness (only a few interatomic spacings) of such a boundary. The boundary of the second type is looser and wider. The proposed discontinuity (looseness) in such a boundary may be caused by the presence of residual irregularities at the joined surfaces.

All the observed types of transition regions (boundaries) between the Ti_50_Ni_25_Cu_25_ and Fe_50_Ni_33_B_17_ layers prevent noticeable interdiffusion of the elements. Within the measurement error, the chemical compositions of the deformed layers correspond to their initial compositions. This suggests that the consolidation of materials occurs by their joint severe plastic deformation, upon which the fragments of the Fe_50_Ni_33_B_17_ and Ti_50_Ni_25_Cu_25_ amorphous alloys strongly approach each other. The overlap (collectivization) of the valence electrons of the neighboring atoms causes the formation of new chemical bonds. The action of interatomic interaction forces leads to the connection of heterogeneous layers and formation of the MMC. There are also published papers that indirectly confirm our assumptions about the leading role of severe plastic deformation upon HPT [38,39,40]. It is impossible to categorically deny the possible occurrence of diffusion processes, but, in our case, they are apparently of secondary importance and can occur in a very narrow region of several interatomic spacings in thickness.

## 5. Conclusions

1. The possibility of MMC formation upon room-temperature HPT of two different alloys, Ti_50_Ni_25_Cu_25_ and Fe_50_Ni_33_B_17_, is shown. At the same time, the alloys undergo opposite structural phase transformations when they are tested separately under the same HPT conditions.
2. It has been established that, upon joint HPT, the Fe_50_Ni_33_B_17_ alloy remains amorphous, whereas the Ti_50_Ni_25_Cu_25_ alloy undergoes a transition from the crystalline to the amorphous phase. As a result, starting from the degree of deformation to *n* ≥ 2, two amorphous Ti_50_Ni_25_Cu_25_ and Fe_50_Ni_33_B_17_ alloys are cooperatively deformed and consolidated into the MMC.
3. Upon the consolidation of the two amorphous alloys, the following types of transition regions between different amorphous phases were observed by transmission electron microscopy: (1) a loose transition region 1–10 nm thick and (2) a narrow, almost invisible transition region.
4. The experimentally determined chemical compositions of the Ti_50_Ni_25_Cu_25_ and Fe_50_Ni_33_B_17_ alloy fragments in the regions of consolidation after HPT correspond to the initial compositions of the alloys. Therefore, all types of transition zones between the layers prevent significant interdiffusion of chemical elements between the Fe_50_Ni_33_B_17_ and Ti_50_Ni_25_Cu_25_ layers.
5. The experimental results indicate that the amorphous layers of the alloys under study are consolidated by their joint severe plastic deformation upon HPT.

## Data Availability

The raw/processed data required to reproduce these findings cannot be shared at this time as the data also forms part of an ongoing study.
